# How Does an Environmental Information Disclosure of a Buyer’s Enterprise Affect Green Technological Innovations of Sellers’ Enterprise?

**DOI:** 10.3390/ijerph192214715

**Published:** 2022-11-09

**Authors:** Chenxi Zhang, Shanyue Jin

**Affiliations:** College of Business, Gachon University, Seongnam 13120, Korea

**Keywords:** environmental information disclosure, green technological innovations, financing constraints, public concern, internal control

## Abstract

With rapid economic development, green technological innovations are playing an important role in the sustainable development of enterprise. When the public is concerned about the environment and values environmental information disclosures, it makes enterprise fulfill their environmental responsibilities. In a supply chain, buyer enterprise’ environmental information disclosures have a spillover effect on seller enterprise’ investment decisions. This study investigates the relationship between environmental information disclosures by buyers and green technological innovations of sellers, furthermore, it analyzes the mechanism of this relationship. For this purpose, this study conducts a fixed regression analysis using the data pertaining to A-share listed companies in China from 2009 to 2019. The results show that buyer firms’ environmental information disclosures can significantly promote the green technological innovations of seller enterprise. Furthermore, financing constraints, public concern, and internal control play a mediating role between buyer firms’ environmental information disclosures and seller firms’ green technological innovations. This study reveals several paths through which environmental information disclosures of buyers influence sellers’ green technological innovations in supply chains.

## 1. Introduction

Even though China’s economy has grown rapidly in recent years, its high-input, high-emission, and high-consumption development approach has caused serious environmental pollution problems. According to Zhang, Xing [1], China ranks 120th out of the 180 countries and regions involved in environmental protection assessments. This is a far cry from China’s global economic ranking, indicating a contradiction between environmental protection and economic growth in China [2]. To resolve this contradiction, enterprise must focus on green technological innovations. Green technological innovations by companies play a pivotal role in achieving the twin objectives of improving environmental quality and promoting sustainable economic development [3].

Green technological innovation is different from general innovations in that it is more systematic and complex, requires a large amount of financial and human resources, and involves greater risk and uncertainty. Therefore, external stakeholders of companies, including the capital market and public, need to provide sufficient motivation to support green development [4].

Enterprise are the largest contributors of both green technological innovations and environmental problems [5]. In the past, China’s crude economic development led to increasingly prominent environmental problems, such as resource shortages and ecological environment issues, and environmental protection has become increasingly important [6]. The external stakeholders’ desires and demands to access environmental information of companies have gained prominence [7]. Especially, as competition between supply chains intensifies, buyers, as important stakeholders of business, can have an important influence on business investment decisions [8]. Companies in the supply chain are interdependent in their production and operation, and they are important stakeholders of each other. Under market mechanisms, demand is usually preceded by supply, and information in the supply chain is transmitted from the bottom to the top. Therefore, seller enterprises not only rely on their own information when making investment decisions, but also consider the relevant information disclosed by buyer enterprises. The buyer’s environmental information disclosure behavior impacts the seller’s business and strategy in many ways [9].

Environmental information disclosed by companies is given due consideration by rating agencies and bond market participants, and companies that provide high-quality environmental information to their buyers have to pay lower risk premiums and lower costs for equity capital [6]. The demand from buyers to incorporate environmentally responsible practices can promote the mutual supervision of economic agents and improve the overall environmental performance of an industrial chain [10]. Such a demand drives supply, triggering a series of economic activities.

The buyers’ demand for environmental information disclosures can directly promote green technological innovations of seller businesses. Further, it can alleviate the degree of financing constraints and improve the quality of internal controls in seller enterprise through information spillover. In addition, reputation spillover enhances three mediated paths of the public attention of seller enterprise.

Green technological innovations by seller enterprise involve great risk and uncertainty and require significant capital investments. Therefore, relying on endogenous corporate financing alone is insufficient to support innovation activities, as exogenous financing is also required [11]. In the capital market, external investors with inferior information have difficulty predicting future returns.

In addition, the degree of information asymmetry is large, making investments prone to moral hazard and adverse selection problems, such as risk concealment. Investors often respond to risks through risk premiums or investment cuts, which makes companies face financing constraints, such as high financing costs [12]. However, when an enterprise discloses information, it has direct or indirect effects on other players in an industrial chain, called the “spillover effect” [13].

Therefore, investors not only rely on the information disclosed by an enterprise itself but also pay attention to the information disclosed by other supply chain enterprise in their investment decisions [14]. Firms with good environmental performance have stronger incentives to disclose information and distinguish themselves from those with poor environmental performance. Firms with high-quality buyer-side environmental information disclosures can send good signals to the market, so that the public can better understand the fulfillment of their corporate environmental responsibility [10].

According to the spillover effects of social responsibility reputation, corporate reputation information has the characteristics of public goods and can generate positive externalities. Socially responsible behavior of one enterprise can influence the behavior of other enterprise with which it has close relationships. Therefore, environmental information disclosure behavior of buyer enterprise can enhance the reputation of seller enterprise by making the latter face the public supervision of their energy consumption and pollution in their production and operation processes [5].

The paper investigates whether environmental information disclosures by buyer enterprises influence green technological innovations of seller enterprises and further analyzes the mechanism of this relationship.

Based on the analysis of the literature and real-world context, we constructed a study with Chinese listed companies in Shanghai and Shenzhen stocks from 2009 to 2019. From a supply chain perspective, based on information disclosure spillover effects. Empirically testing the effect of environmental information disclosure of buyer firms on the level of green technology innovation of seller firms, the results indicate that environmental information disclosure of buyer firms is considered to promote green technology innovation of seller firms. The study further explores the influence mechanism of its connotation using the intermediary effect. The empirical study found that the environmental information disclosure of buyer firms can promote the green technology innovation of seller firms by alleviating the financing constraints of seller firms.

This study analyzes the spillover effects of buyer firms’ environmental information disclosures in a supply chain and reveals the important path that a buyer enterprise’s environmental information disclosure takes to affect seller firms’ green technological innovations through the latter’s financing constraints, public attention, and internal controls. It provides an important theoretical reference for expanding green technological innovations of seller firms and achieving sustainable development.

Most existing studies have focused on the effects of command-and-control and market-incentive environmental regulations on corporate micro-entities. Few papers have verified the impact of environmental information disclosure on corporate green technology innovation from the perspective of public participation-based environmental regulation. This study is an important theoretical reference for expanding the research paradigm of corporate green technology innovation. This study analyzes the spillover effects of environmental information disclosure in the supply chain based on the supply chain bars of the top five customers of listed companies. It reveals the special and important path that environmental information disclosure affects corporate green technology innovation through the supply chain, enriches the literature view of information disclosure spillover effect, and can provide reference for corporate supply chain management.

The structure of the study is as follows: the introduction summarizes the background and significance of the study in the first section. The theoretical background and hypotheses are conducted in the second section. The third section concludes the variable design and the research model design. In the fourth and fifth section, we show the results of empirical analysis and robustness tests. Finally, the results of data analysis are discussed, and relevant policy recommendations are suggested.

## 2. Theoretical Background and Hypotheses

### 2.1. Environmental Information Disclosure

With increasingly prominent environmental problems and rapid economic development, China’s environmental regulatory tools have gradually changed from a command-and-control model to a “four-dimensional” environmental regulatory policy system of command-and-control, market incentives, public participation, and voluntary action [15]. Environmental governance in western developed countries has generally entered the stage of environmental information disclosure, and environmental information disclosure policy has become an important tool of environmental control for the U.S. government [3]. As a type of public participation in environmental regulation, environmental information disclosure means giving the public information about the positive and negative effects of an organization’s activities on the environment over a certain period. By reducing the information asymmetry between enterprise and the outside world, it relies on social supervision and investor decisions to influence corporate behavior and promote corporate environmental governance [16]. Environmental information disclosure in China is different from general information disclosure, which is a voluntary disclosure with the characteristics of initiative and discretion. In China, environmental information disclosure is a way for enterprise to disclose environmental accounting information to the public solely for their own purposes and possibilities. enterprise consider their external image, reasonable tax avoidance, and public recognition of managers’ environmental ethics and social responsibility to disclose environmental information [17].

Corporate environmental information disclosure has different motivations, such as industry differences and external institutional pressures, company characteristics, and stakeholder influence, which affect the choice and quality of environmental disclosure, based mainly on two different theories: economic disclosure theory and socio-political theory [18]. The economic disclosure theory is based on the signaling theory, which argues that the higher the level of environmental information disclosure, the better the environmental performance of a firm, and that firms choose to actively disclose environment-related information to obtain extraordinary returns [19]. Firms with high environmental performance indicate their environmental advantages to stakeholders through environmental disclosures to differentiate themselves from other firms to gain a competitive advantage, whereas firms with poor environmental performance have a lower propensity to disclose environmental information [20]. In contrast, social-political theory suggests that companies passively disclose corporate environmental information in order to meet the requirements of the policy regime and to demonstrate that they have fulfilled their “social contract” to avoid legitimacy constraints and the threat of legal action [21]. Therefore, companies with poor environmental performance are more willing to actively disclose environmental information in order to deal with the negative social impacts of poor environmental performance.

Firms in a supply chain are interdependent in terms of their production and operations; therefore, they are important stakeholders of each other. Under the market mechanism, demand is usually preceded by supply, and information in a supply chain is transmitted from the bottom to the top; thus, seller enterprise not only rely on their own information when making investment decisions, but also consider the information disclosed by buyer enterprise. The environmental information disclosure behavior of buyer firms has many impacts on the operation and strategy of seller firms [20].

Corporate environmental information disclosure can have different economic consequences, and relevant studies have focused on the effects of environmental information disclosure on corporate value, environmental performance, and stakeholders. By improving the level of environmental information disclosure, companies can demonstrate their effective environmental risk management capabilities and corporate development prospects, can promote their environmental performance, enhance investor confidence [22], help companies increase their share prices in the short term [23], and increase corporate value by reducing equity capital and increasing expected cash flows [24]. Environmental information disclosure shows that companies actively fulfill their environmental responsibilities and that there are spillover effects of environmental responsibility that can cross corporate boundaries and have an impact on other companies [25]. At the same time, the public and other stakeholders’ perceptions of an enterprise’s reputation for environmental responsibility are also influenced by the behavior of related enterprise, and spillover is mainly realized through contagion and contrast effects [26]. Environmental information disclosure is an important channel for companies to convey corporate environmental behavior to the public, investors, customers, suppliers, and other stakeholders, and to improve social recognition, which can reduce the important impact of information asymmetry on stakeholders’ decision-making. Environmental information disclosed by companies receives attention from rating agencies and bond market participants [27], and companies with good-quality environmental information disclosure have a lower risk premium and can obtain a lower cost of equity capital [28]. Demand drives supply and triggers a series of economic activities, and the demand for downstream environmental responsibility can promote mutual supervision of economic agents in the chain and the improvement of the overall environmental performance of the chain [2].

Green technology innovation is a broad concept, also known as sustainable innovation, eco-innovation, environmental innovation, etc. Green technology innovation generally refers to innovations that follow ecological laws and can achieve resource conservation, pollution reduction and environmental improvement. Regarding the research on the motivation of green technology innovation, the existing literature is mainly based on the perspectives of strategic management, environmental economy, and industrial organization [29]. The strategic management perspective focuses on the internal factors that influence the implementation of green technology innovation strategies, and green technology innovation is influenced by organizational characteristics and executive support, etc. R&D investment is closely related to a company’s ability to innovate green technology, which, in turn, has a positive effect on green technology innovation performance [30]. In addition, top management support or environmental awareness significantly and positively affects firms’ green technology innovation practices [31], a strong and independent board of directors can monitor managers, reduce their short-sighted behavior, and promote green technology innovation, and board governance can positively regulate the relationship between environmental regulation and green technology innovation [32].

The information asymmetry theory refers to the fact that the two parties to a transaction do not have equal access to information. The party with more information may take advantage of the information for its own benefit in the market to the detriment of the other party. Environmental information disclosure can improve the transparency of environmental information and effectively reduce the information asymmetry between enterprises and stakeholders [33].

As public information, a buyer’s disclosure is subject to external supervision and has higher credibility, which can reduce the information search and decision cost of seller firms, thereby lowering the uncertainty risk caused by information asymmetry [34]. Environmental information disclosed by buyer firms enables seller enterprise to gain a more comprehensive understanding of buyer firms’ environmental performance and accurately predict their green needs.

There are two channels for seller companies to obtain major buyer information: one is through public information, such as annual reports and social responsibility reports publicly disclosed by companies. Secondly, through private information, enterprise can obtain information on buyer’s demand and buyer’s order in the process of cooperation and communication with buyers [35]. Compared with private information, the seller firm’s investment decision depends on the expected return on investment projects. Buyer enterprise’ demand preferences, as the main source of sales revenue, are the target of sellers’ attention when assessing the expected return on their investment projects [36]. Buyer firms that actively disclose environmental information signal that they are more environmentally conscious, and seller firms that prefer green behaviors for environmental sustainability implement green practices that meet buyer firms’ environmental expectations to prevent market leakage [37].

By obtaining the environmental information disclosed by buyer firms, a seller firm can accurately assess their business risk, default risk, regulatory risk, and future business conditions, which enhances the seller firm’s incentive to strengthen its green technology innovation efforts to meet buyers’ demand [14]. The managers of the seller enterprise believe that the current customer relationship will bring economic benefits to enterprise out of good expectations of the buyer’s future.

Seller enterprise’ green technological innovations can improve their own strengths, enhance productivity, and provide buyers with higher quality and cleaner products and services while reducing environmental pollution, and buyers who disclose environmental information prefer to purchase from companies with green technological innovations [38]. To achieve long-term stable cooperation with buyers, sellers have strong incentives to implement green technological innovation investment strategies to maintain or even deepen this cooperative relationship. Based on the above analysis, we propose Hypothesis 1:

**Hypothesis** **1.** *Environmental information disclosure by buyer firms can promote green technological innovations by seller firms.*

### 2.2. Mediating Role of Financing Constraints

Green technological innovations by seller-side firms involve significant risks and uncertainties and require considerable capital investments. Therefore, firms must seek exogenous financing as well because relying on endogenous financing alone is insufficient.

However, external investors, who are at an information disadvantage in the capital market, have difficulty predicting future returns of firms. This increases the cost of financing for firms and limits the amount of financing. As a result, firms may face financing constraints that seriously limit their enthusiasm for green technological innovations [39]. That said, information disclosed by enterprise have direct and indirect effects on other players, which is known as the “spillover effect” of information disclosure in a supply chain [40].

Therefore, investors while making investment decisions not only rely on the information disclosed by an enterprise itself, but also pay attention to the information disclosed by other supply chain enterprises. Environmental information disclosures by buyer enterprise can reduce the information asymmetry between investors and seller enterprise, enhance investors’ confidence, expand the external financing channels of seller enterprise, and ultimately provide financing support for green technological innovations [41].

Buyers’ environmental information disclosures act as incremental information for firms and reduce the information asymmetry between sellers and external investors [42]. According to stakeholder and signaling theories, a buyer’s environmental disclosures can serve as a potential channel for investors to obtain information about seller enterprises, provide investors with more incremental information, signal the development status of seller enterprises to investors, and reduce the information asymmetry between seller enterprises and external capital suppliers [43].

Investors can objectively assess the environmental risks and investment value of seller enterprise by analyzing the environmental information disclosed by buyer enterprise, which can help them evaluate the financial and business performance of seller enterprise, thus reducing the risk premium required due to uncertainty and lowering the cost of equity financing for such enterprise [44].

In addition, a buyer enterprise helps banks assess the operating conditions of other enterprise in a supply chain by providing environmental information, thereby reducing information asymmetry, giving full play to the linkage of green credit in a supply chain, and helping seller enterprise reduce debt financing costs [45]. Reduction in the external financing cost makes it easier for seller enterprise to obtain funds to support their green technological innovations [12].

Buyers of environmental information disclosure have a stronger incentive to monitor corporate behavior, alleviate agency problems, and increase investor confidence [46]. For managers due to the strong uncertainty and high risk of green technology innovation, and it does not have a significant contribution to short-term performance, but rather has a negative impact. Therefore, it will have a negative effect on the performance appraisal faced by managers, so they will have a relatively negative attitude towards the implementation of green technology innovation strategy and may have opportunistic behavior in the process of green technology innovation implementation [44]. Buyer firms, as one of the important stakeholders of the company, have reputational mechanisms and cross-regulatory roles that can influence the business activities of the company in all aspects, especially in the Chinese market where overcapacity prevails, and large customers are more likely to represent a trust contract. The environmental performance of sell-side firms can influence the market’s evaluation of buy-side firms [47]. Thus, buyer firms that disclose environmental information can discourage opportunistic managerial behavior and reduce information and agency costs for investors in seller firms [48]. By disclosing environmental information on the buyer side, firms receive more generous financial covenants, which, in turn, reduce the cost of external financing to ease financing constraints.

Environmental information disclosures by buyers can effectively improve the external financing ability of seller enterprise through the information spillover effect. This effect can overcome the financing constraints of innovations, enhance the short-term risk resistance of enterprise, and help them implement a green technological innovation strategy. Creating necessary conditions to encourage enterprise to increase research and development (R&D) investments to enhance the level of green technological innovations [49]. Based on the above analysis, we propose Hypothesis 2:

**Hypothesis** **2.** *Environmental information disclosure by buyer firms promotes green technological innovations by seller firms by alleviating the latter’s financing constraints.*

### 2.3. Mediating Role of Public Attention

Firms with good environmental performance have a stronger incentive to disclose information that distinguishes them from those with poor environmental performance. Firms with high-quality buyers’ environmental information disclosures can send positive signals to the market so that the public can better understand how these firms fulfill their environmental responsibilities, thereby enhancing corporate reputation [50].

According to the spillover effect of social responsibility reputation, corporate reputation information have characteristics of public goods and can generate positive externalities. The socially responsible behavior of one enterprise can influence the social responsibility reputation of other enterprise with which it has close relationships. Therefore, environmental information disclosure behavior of buyer enterprise can enhance the environmental responsibility reputation of seller enterprise by making seller enterprise face the public supervision of their energy consumption and pollution in their production and operation processes [5].

Increased public attention forces companies to innovate green technologies. Due to the increasingly challenging living environment, public awareness of environmental protection is gradually increasing and consumer attitudes are changing, as they are becoming more aware of environmentally friendly products and are willing to pay for them [51]. When the public is more concerned about the environmental performance of enterprise, enterprise need to consider environmental protection in their investment decisions; besides, energy consumption and pollution in their production and operations must face public supervision for assessment of their reputation [52]. The public may also put pressure on the government to strengthen its intervention in the environmental protection behavior of enterprise [53]. This is the reason why enterprise choose green technological innovations to reduce the negative impact of their products on the environment.

The theory of information asymmetry refers to the inequality in the possession of information between two parties to a transaction. The party with more information will have more initiative and may take advantage of the information for its own benefit in the market to the detriment of the other party, which may lead to adverse selection and moral hazard problems. This is also the reason why the principal-agent problem arises [54]. Environmental information disclosure, as a demonstration of the practice of corporate environmental responsibility, can improve the transparency of environmental information. It can effectively reduce the information asymmetry between enterprises and stakeholders as well as the public, make stakeholders know more accurately and clearly the fulfillment of corporate environmental responsibility, and enhance the public’s confidence in enterprises [55]. Environmental information disclosure reflects the enterprise’s initiative in energy saving and emission reduction, providing green products, conveying the enterprise’s operation status and good environmental management signals to the outside world, and helping to reduce the seller’s enterprise’s search and decision cost for information [56].

Reputation theory believes that reputation is an intangible asset of an enterprise, which comes from the public’s emotional attraction to the enterprise and is based on the enterprise’s daily behavior, bringing more public attention and consumer preference, helping the enterprise to establish a competitive advantage in the market and increase its market share. Environmental information disclosure can send a signal that a company is actively fulfilling its environmental responsibilities, which, in turn, can enhance its reputation [57]. In addition, green technology innovation can also help companies maintain or enhance their reputation and gain a higher green premium by winning public recognition and support for their products [58].

Public attention, as an important component of external corporate governance, generates firm-related information in the capital market, greatly alleviates the principal-agent problem and information asymmetry, enhances investor confidence in corporate green development, and enables seller firms to have sufficient capital for green technological innovations [6]. In addition, seller enterprise can use green technological innovations to increase their confidence in green development. Further, through green technological innovations, seller enterprise can shape their green image, reduce the negative impact of their production and operational activities on the environment, and gain a good reputation. To maintain a good reputation, managers strengthen corporate governance to reduce opportunism and show positive environmental behavior, which stimulates their desire to strengthen green technological innovations in seller enterprise [59]. Thus, Hypothesis 3 is proposed below based on the above analysis.

**Hypothesis** **3.** *Environmental information disclosures by buyer firms promote their green technological innovations by enhancing the public attention of seller firms.*

### 2.4. Mediating Role of Internal Controls

Internal control systems cover the overall operational mechanisms of a firm and ensure that it operates rationally toward increasing its value [60]. This process involves not only the production, consumption, and operational procedures of an enterprise, but also balances the relationship between the enterprise and its external environment [13]. Internal control, as an important component of an enterprise’s internal governance, has a supervisory effect, as it greatly alleviates the principal-agent problem and information asymmetry, enhances investor confidence in enterprise’ green development, and enables firms to have sufficient capital for green technological innovations [61].

The principal-agent theory states that the separation of ownership and operations of a company leads to a conflict of interests between shareholders and management, and information asymmetry allows the management to pursue its own interests; as a result, the management may act opportunistically to the detriment of shareholders [60]. Compared with shareholders, the management is more inclined to sacrifice the long-term interests of a firm to seek short-term profits for its own sake, thus influencing the future investment decisions of a firm [62].

Firms with high-quality environmental information disclosures by buyers can send positive signals to the market. Information disclosures can be transmitted vertically along the supply chain, having spillover effects [63]. Favorable internal systems can effectively control the actions of managers, thus ensuring that more reliable and accurate financial information is disclosed, thereby reducing principal-agent problems [63]. When a seller cannot effectively satisfy buyers’ demand, its market competitiveness is reduced, and it is likely to lose its market share. When a buyer has better internal controls, the seller must enhance its own internal control requirements to meet the buyer’s product and reputational needs [64]. Therefore, Hypothesis 4 is proposed as follows:

**Hypothesis** **4.** *Buyer firms’ environmental information disclosures promote green technological innovations by improving the quality of seller firms’ internal controls.*

**Figure 1:**
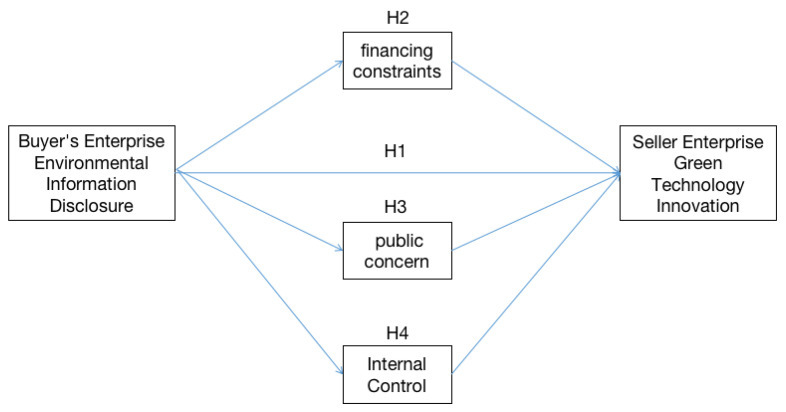
Study Model.

Figure 1 is the research model of the study.

## 3. Research Methods

### 3.1. Data and Samples

In view of China’s environmental information disclosure policy enacted in 2007 and the global financial crisis in 2008, this study conducted research on Chinese listed companies from 2009 to 2019. In this study, the data of top five customers of listed companies and environmental information disclosure of buyer companies are obtained from CSMAR (China Stock Market and Accounting Research Database). The data of green technology innovation of listed companies are obtained from the green patent database of CNRDS (China Research Data Service Platform). The finance and insurance samples were excluded from the industry data.

Internet search index data were obtained from the Internet search index database of the China Research Data Service Platform. For data processing, the samples of financial and insurance industries were excluded from this study, and the samples with missing values were excluded from the data. To reduce the effect of outliers, the variables were winsorized at the upper and lower 1% levels (Environmental information disclosure and corporate age excluded). In addition, continuous variables are shown as logarithms to reduce the heteroscedasticity of the disturbances.

### 3.2. Definition and Measurement of Variables

Green technology innovation, also known as sustainable innovation, eco-innovation, environmental innovation, etc. It generally refers to innovations that follow ecological laws and can achieve resource conservation, pollution reduction, and environmental improvement [29]. Enterprises can reduce costs and expand production to gain competitive advantage through technological innovation [1].

Invention patent refers to innovative technical achievements or ideas that can be directly applied in production. A utility model patent is defined that the innovative design of the appearance and structure of a product, which is generally innovative only on the basis of the original product. Utility model patents do not involve the reform of product manufacturing principles and are less innovative than invention patents. Therefore, the measurement of green technology innovation in this study use green patents and green invention patents [29].

Green technological innovations consider resource conservation and pollution reduction in all aspects of product design, production, use, and recycling, and include both process- and product-related innovations. The explanatory variables in this study are corporate green patents (TGreen) and corporate green invention patents (IGreen) of listed companies. TGreen is the logarithm of the number of green patent applications applied by enterprise in the current year plus one; IGreen is the logarithm of the number of green invention patent applications applied by enterprise in the current year plus one [65]. IGreen is the logarithm of the number of green patent applications filed by enterprise in the same year.

The explanatory variable in this study is the level of environmental information disclosures (EDI) by buyer firms [63]. The specific judgment criteria were as follows: no environmental information disclosure was scored as 0, qualitative environmental information disclosure was scored as 1, and quantitative environmental information disclosure (quantitative and monetary) was scored as 2. The scores of the selected environmental information disclosure indicators are summed up to obtain the level of environmental information disclosure by enterprise as follows:

$${\mathrm{EDI}}_{i,t}=\sum_{i=1}^{n}{\mathrm{edi}}_{\mathrm{ijt}}$$

${\mathrm{EDI}}_{i,t}$ is the sum of the scores of environmental information items disclosed by company i in year t. ${\mathrm{edi}}_{\mathrm{ijt}}$ is the score of company i on j environmental information items in year t. ${\mathrm{EDI}}_{i,t}$ A higher score indicates a higher quality of environmental information disclosure by a company.

The mediating variables in this study are financing constraints (SA), public attention (Att), and internal controls (ICI) [48,66]. To calculate the buyer financing constraint (SA), the SA index constructed by Hadlock and Pierce (2010) was used to measure the overall financing constraint status of listed companies’ SA index, which is calculated as follows:

$$\mathrm{SA}=-0.737\times \mathrm{size}+0.043\times {\mathrm{size}}^{2}-0.040\times \mathrm{age}$$

where size is the natural logarithm of firm size. Age is a firm’s establishment time.

Public attention (Att) was calculated using a web search index. Internet users usually use search engines to obtain information. When users use search engines to search for a company’s name, their concern about the company is directly displayed. Therefore, the Internet search index can reflect the Internet users’ sentiment and company search fever to a certain extent. So, in this study, the search values of keywords of a company’s stock code, full name, and abbreviation were summed up to obtain the company. Therefore, in this study, the Internet search index was obtained by summing up the search values of keywords of enterprise stock code, enterprise full name, and enterprise abbreviation [64]. In this study, the internet search index was obtained by summing up the enterprise stock code, enterprise name, and enterprise abbreviation to indicate the social public attention of the enterprise [63].

Regarding the mediating variable of internal control (ICI), the Diebold Index was selected and was logarithmically treated. The Diebold Index is a basic internal control index designed based on the degree of achievement of the five major objectives of internal control compliance: reporting, asset security, operation, and strategy. Internal control deficiencies were used as a correction variable to amend the basic internal control index, resulting in a comprehensive index that reflects the level of internal control and risk control ability of listed companies [67].

The control variable in this study is firm age (Age), which usually represents a firm’s maturity, and the logarithm of the number of years a firm has been listed is used to measure firm age [68]. When it comes to the size of a firm (Size), it is generally believed that larger firms make more stable R&D investments to maintain their level of technological development, and the logarithm of a firm’s total capital at the end of a year is selected to measure the firm’s size [62]. The higher the value, the greater the debt servicing pressure on an enterprise; a significant debt servicing pressure on an enterprise is not conducive for its green technological innovations [66].

The ratio of net cash flow is higher, the more cash an enterprise can use for investment, and the higher the ability to resist risk, which helps the implementation of green technological innovations [68,69,70]. The ratio of total fixed assets to the number of employees was chosen as a logarithmic measure of capital intensity.

In modern enterprise, independent directors can avoid the erosion of the interests of major shareholders in internal governance and influence the investment decisions of enterprise; the ratio of independent directors in the board of directors of an enterprise is defined as independence ratio (Indr) [31].

As green technological innovations have strong uncertainty and high risk, they may not be suitable to improve the management’s short-term performance, so management incentives can influence their motivation to innovate green technologies. Therefore, the ratio of management shareholding to total corporate equity was selected to measure management incentives (Share) [32]. The ratio of annual R&D investments to operating revenue was chosen to measure R&D investment [40]. The external financial dependence index (EFD) controls for financial dependence indicators to better analyze the role of financing constraints. Selecting the firm’s capital expenditures minus cash [1].

Definitions of the variables are explained in Table 1.

### 3.3. Research Model

To analyze the impact of buyer firms’ environmental information disclosures on seller firms’ green technological innovations, drawing on Gao and Jin [71], the following panel econometric model was developed in this study:

(1) $${\mathrm{Green}}_{i,t}=\alpha_{0}+\alpha_{1}{\mathrm{EDI}}_{i,t-1}+{\delta \mathrm{Controls}}_{i,t}+\mathrm{Year}+\mathrm{Industry}+\varepsilon$$

Model (1) tests the impact of environmental information disclosures of buyer firms on green technological innovations of seller firms, that is, Hypothesis 1. Considering that the environmental information of a buyer, disclosed at the end of the current year, affects the green technological innovations of seller firms only in the next year, the level of environmental information disclosures by buyer firms in period t−1 was selected as the explanatory variable in this study. The subscript t in the model denotes the year, subscript i denotes the firm, ${\mathrm{Green}}_{i,t}$ denotes the level of green technological innovations of firm i in year t, ${\mathrm{EDI}}_{i,t-1}$ denotes the level of environmental information disclosures by a buyer firm in the previous year, ${\mathrm{Controls}}_{i,t}$ denotes the control variables, $\alpha_{0}$ is the constant term of the regression model, and ε is the random disturbance term of the model. When $\alpha_{1}$ > 0, it indicates that the environmental information disclosures of buyer enterprise can improve the level of green technological innovations of seller enterprise. However, when $\alpha_{1}$ < 0, the opposite is true.

To further test whether financing constraints of seller firms play a mediating role in the relationship between buyer-firms’ environmental information disclosures and seller-firms’ green technological innovations, we add financing constraints of seller-firms to the benchmark model to test Hypothesis 2.

(2) $${\mathrm{SA}}_{i,t}=\alpha_{0}+\alpha_{1}{\mathrm{EDI}}_{i,t-1}+{\delta \mathrm{Controls}}_{i,t}+\mathrm{Year}+\mathrm{Industry}+\varepsilon$$

(3) $${\mathrm{Green}}_{i,t}=c_{0}+c_{1}{\mathrm{EDI}}_{i,t-1}+c_{2}{\mathrm{SA}}_{i,t}++{\delta \mathrm{Controls}}_{i,t}+\mathrm{Year}+\mathrm{Industry}+\varepsilon$$

Performing the test of the regression equation between the independent and mediating variables, that is, using Model (2) to test that ${\mathrm{SA}}_{i,t}$ denotes the seller firm’s financing constraint. If coefficient $\alpha_{1}$ is significant, it means that environmental information disclosures of buyer firms significantly alleviate the financing constraints of seller firms. However, it does not indicate the existence of a mediating effect and needs to be continued in the next step of the test. If the coefficient of Model (3), $c_{2},$ is significant, it indicates that the mediating effect is significant.

To test Hypothesis 3, that is, whether public concern for seller-firms plays a mediating role in the relationship between environmental information disclosures of buyer-firms and green technological innovations of seller-firms, public concern for seller-firms was added to the baseline model.

(4) $${\mathrm{Att}}_{i,t}=\alpha_{0}+\alpha_{1}{\mathrm{EDI}}_{i,t-1}+{\delta \mathrm{Controls}}_{i,t}+\mathrm{Year}+\mathrm{Industry}+\varepsilon$$

(5) $${\mathrm{Green}}_{i,t}=c_{0}+c_{1}{\mathrm{EDI}}_{i,t-1}+c_{2}{\mathrm{Att}}_{i,t}+{\delta \mathrm{Controls}}_{i,t}+\mathrm{Year}+\mathrm{Industry}+\varepsilon$$

The test of the regression equation between the independent and mediating variables is performed using Model (4) to test that ${\mathrm{Att}}_{i,t}$ denotes the public attention of seller-firms. If coefficient $\alpha_{1}$ is significant, it means that a buyer’s environmental information disclosures significantly increase the public attention for a seller firm. However, it does not indicate the existence of a mediating effect, and the next test needs to be conducted. If the coefficient of Model (5), $c_{2},$ is significant, it indicates that the mediating effect is significant.

To test Hypothesis 4, that is, whether seller firms’ internal controls play a mediating role in the relationship between buyer-firms’ environmental information disclosures and seller-firms’ green technological innovations, seller firms’ internal controls were added to the benchmark model.

(6) $${\mathrm{ICI}}_{i,t}=\alpha_{0}+\alpha_{1}{\mathrm{EDI}}_{i,t-1}+{\delta \mathrm{Controls}}_{i,t}+\mathrm{Year}+\mathrm{Industry}+\varepsilon$$

(7) $${\mathrm{Green}}_{i,t}=c_{0}+c_{1}{\mathrm{EDI}}_{i,t-1}+c_{2}{\mathrm{ICI}}_{i,t}+{\delta \mathrm{Controls}}_{i,t}+\mathrm{Year}+\mathrm{Industry}+\varepsilon$$

Using Model (6) to test the regression equation between the independent and mediating variables, ${\mathrm{ICI}}_{i,t}$ denotes seller firms’ internal controls. If coefficient $\alpha_{1}$ is significant, it indicates that the disclosure of environmental information by buyer firms significantly improves the internal controls of seller firms. However, it does not indicate the existence of a mediating effect and needs to be continued in the next step of the test. While testing Model (7), if coefficient $c_{2}$ is significant, it indicates that the mediating effect is significant.

The original hypothesis *p* < 0.05 was rejected after the Hausman test; therefore, the fixed effects model was used in this study [72].

## 4. Results

### 4.1. Descriptive Statistics

The mean value of green patents (TGreen) is 0.600 and the minimum value is 0, which indicates that the enterprise does not have overall awareness of green technological innovations. The standard deviation is 0.910, which indicates that the level of green technological innovations among the selected sample varies greatly. The mean value of green invention patents (IGreen) is 0.393, which is higher than that of the green utility model patent (0.364), indicating that enterprise prefer green product innovations to green process innovations and are more motivated to apply for green invention patents. The maximum value of environmental information disclosures (EDI) is 14.56, the minimum value is 0, and the standard deviation is 1.986, indicating that the quality of environmental information disclosures by sample buyer enterprise vary. The mean value of EDI, 1.024, is at a low level, indicating that the overall quality of environmental information disclosures by buyer enterprise is not high.

The financial indicators and governance variables are within normal levels. The mean value of gearing (Lev) is 0.395 with a standard deviation of 0.249, and the mean value of return on net assets (Roa) is 0.046 with a standard deviation of 0.102, indicating that the strength of the sample companies differs more significantly. The maximum value of R&D investment (RD) is 23.68, the minimum value is 0, and the standard deviation is 2.286, which shows a significant difference in the importance given to R&D investments by different companies. Table 2 presents the results.

### 4.2. Correlation Analysis

Table 3 shows that the correlation between buyer enterprise’ environmental information disclosures and seller enterprise’ green technological innovations is significant at the 5% significance level, indicating a significant positive relationship between these two variables, which to some extent verifies Hypothesis 1. The mean value of VIF is less than 1.5, which shows that there is no multicollinearity [13].

### 4.3. Analysis Results

To test Hypothesis 1, that is, the effect of environmental information disclosures by buyer firms on corporate green technological innovations, we perform a regression analysis corresponding to Model 1 in Table 4.

As shown in Table 5, the regression coefficients of environmental information disclosures (EDI) are 0.0477 and 0.0524, which are significantly positive at the 5% and 1% levels, respectively, indicating that environmental information disclosures by buyer enterprise can significantly enhance the green technological innovation levels of seller enterprise, supporting Hypothesis 1. Buyer environmental information disclosures have a spillover effect in supply chains, which can significantly influence the green technological innovation behavior of seller enterprise. Sellers can reduce the information asymmetry between buyers and sellers through environmental information disclosed by buyers and accurate understanding of buyers’ green preferences for products and services. To improve their competitiveness or maintain a cooperative relationship with buyers, sellers can adopt green technological innovations.

Table 6 reports the regression results for the mediating effect of financing constraint mitigation corresponding to Model 2. This shows a significant positive contribution of buyer firms’ environmental information disclosures on seller firms’ financing constraints, indicating that buyer firms’ environmental information disclosures (EDI) can effectively alleviate seller firms’ financing constraints (SA). The third and fifth columns show the regression results of Model (3) when the total number of green patents (TGreen) and green invention patents (IGreen) are the explanatory variables, respectively. Buyers’ environmental information disclosures and sellers’ financing constraints are significantly positive at different levels and can considerably contribute to corporate green technological innovations. Therefore, based on the above results, the following analysis of the mediating effect can be made: the coefficients of the explanatory variables on explained variables are all significantly positive, and it can be said that corporate financing constraints have a mediating role in the relationship between buyers’ environmental information disclosures and sellers’ green technological innovations. Therefore, Hypothesis 2 is supported.

According to the information spillover effect, a buyer firm, as an important stakeholder of a seller enterprise, has a significant influence on the business conditions and investment decisions of the seller enterprise. Environmental information disclosed by buyer enterprise can effectively reduce the degree of information asymmetry between seller enterprise and their investors by providing more incremental information for investors to effectively evaluate green-related investments of seller enterprise, enhancing investors’ confidence, reducing risk premium demanded by investors, and alleviating corporate financing constraints.

Table 6 reports the regression results of the mediating effect of public attention enhancement corresponding to Model 4. Buyer-firms’ environmental information disclosures have a significantly positive effect on public attention paid to seller firms at the 1% level, indicating that buyer-firms’ environmental information disclosure (EDI) can effectively enhance seller firms’ public attention (Att). The third and fifth columns show the regression results of Model 5 when the total number of green patents (TGreen) and green invention patents (IGreen) are the explanatory variables, respectively. Buyers’ environmental information disclosures and public attention toward seller firms contribute significantly to corporate green technological innovations. Based on the above results, the following analysis of the mediating effects can be made: the coefficients of the explanatory variables on explained variables are significantly positive. It can be said that the public attention paid to seller enterprise has a mediating effect on the relationship between environmental information disclosures of buyer enterprise and green technological innovations of seller enterprise. Therefore, Hypothesis 3 is verified.

According to the reputation spillover effect, the environmental information disclosure behavior of buyer enterprise can cross the enterprise boundary, effectively enhancing the public attention paid to seller enterprise, and improving the reputation of seller enterprise. When the public attention toward seller enterprise is enhanced, it stimulates the green technological innovation behavior of such enterprise. On the one hand, the public amplifies the impact of various behaviors of an enterprise when the social attention toward the enterprise increases. On the other hand, an increase in public attention makes enterprise obtain higher premiums when they carry out green technological innovations, which, in turn, earns them greater support from the public.

Table 7 reports the regression results for the mediating effect of internal controls’ enhancement corresponding to Model 6. It indicates that environmental information disclosure (EDI) of buyer firms can effectively enhance the internal controls (ICI) of seller firms. The third and fifth columns show the regression results of Model 7 when the total number of green patents (TGreen) and green invention patents (IGreen) are the explanatory variables, respectively. Buyers’ environmental information disclosures and sellers’ internal controls contribute significantly to sellers’ green technological innovations. Based on the above results, the following analysis of the mediating effect can be made: the coefficients of the explanatory variables on the explained variables are significantly positive. It can be said that the internal controls of seller firms have a mediating effect on the relationship between environmental information disclosures by buyer firms and green technological innovations of seller firms. Therefore, Hypothesis 4 is verified.

Seller-firms with high-quality buyer-side environmental information disclosures can send positive signals to the market. According to the information spillover effect, buyer firms’ environmental information disclosure behavior can cross corporate boundaries. When a seller firm with good internal controls can effectively restrain the behavior of operators and managers, it stimulates the green technology innovation behavior of the firm. When a buyer firm has good internal controls, the seller firm also enhances its own internal control requirements to satisfy the buyer’s product and reputation demands.

## 5. Robustness Analysis

To examine the robustness of the empirical results, this study replaces the dependent variable of green technological innovations with the number of green patents granted by a firm in the current year to verify whether the main findings discussed in Section 4 still hold. The number of green patents granted is represented by two variables: the total number of green patents granted (AthrTGreen), and the total number of green invention patents (AthrIGreen) [73]. The number of patents granted was also processed by adding one to the logarithm. The results are presented in Table 8.

The regression results in Table 8 show that when the explanatory variables are the total number of green patents granted (AthrTGreen) and the total number of green invention patents (AthrIGreen), the regression coefficients of buyer firms’ environmental information disclosure (EDI) are 0.0497 and 0.0572, which are significant at the 5% and 1% levels, respectively. This indicates that the environmental information disclosures by buyer firms can positively promote green technological innovations of seller firms, proving that the benchmark results are robust.

## 6. Conclusions

### 6.1. Discussion

The development of green technologies has given rise to a range of new business models. The study found that the development of green technology innovation has reduced the problem of financing dilemmas faced by innovative firms. It further enhances the willingness of enterprise to innovate technology and make technological innovation. Firstly, the results show that green technology innovation fills the gap in traditional areas. It enables enterprise to obtain a more stable flow of funds to support their innovation projects and achieve an optimal allocation of financial resources. Secondly, the results showed that the environmental information disclosure of buyer enterprises can alleviate the financing constraints of seller enterprises through information spillover and enhance the level of green technology innovation of enterprises. At the same time, environmental information disclosure by buyer firms can improve green technology innovation by increasing public attention. Additionally, the environmental information disclosure of buyer enterprises can improve the green technology innovation level of enterprises by improving internal control. It enhances the information identification ability of enterprise, helps them judge the innovation direction and market potential, as well as maintain customers, etc., as well as improves the efficiency of their innovation decisions. In addition, due to the profit-seeking nature of capital, enterprise will focus more on their core innovation competitiveness and concentrate their resources on these innovation activities, with little impact on non-substantial innovations with less economic potential.

Enterprise are the largest subject of green technological innovation and the most important parties involved in environmental problems. Under the increasingly urgent ecological situation, environmental information disclosure, and green technology innovation are attracting increasingly grow attention. We believe that analyzing the mechanisms that affect the green technology innovation of enterprise from the perspective of supply chain stakeholders is more conducive to accelerating the green technology innovation of enterprise. At the same time, the internal governance of enterprise and public participation in the environment play a supervisory and incentive role. This study has important implications for how environmental information disclosure affects green technology innovation.

### 6.2. Conclusions, Implications, and Limitations Section

As various enterprise in the supply chain are working more closely with each other than before, buyer enterprise’ information disclosure behavior can have a vertical spillover effect in supply chains and influence the green technological innovation decisions of seller enterprise. Therefore, this study examines the influence of buyer firms’ environmental information disclosures on seller firms’ green technological innovation level from the supply chain perspective based on the information disclosure spillover effect. For this purpose, the data of Chinese enterprise listed on Shanghai and Shenzhen stock markets from 2009 to 2019 were considered. Moreover, this study explores the influence mechanism of its connotation by using the intermediary effect to reveal the following insights.

First, buyers’ environmental information disclosures have a spillover effect which, through vertical relationships in a supply chain, can significantly promote seller-firms’ green technological innovations. Information disclosed by seller firms can reduce information asymmetry and better predict buyer firms’ demand for green products and services. Moreover, favorable environmental information disclosures by buyer firms can make seller firms have a better expectation of buyer firms’ business conditions. This makes seller firms more willing to achieve long-term cooperation with buyer firms and motivates seller firms to meet buyer firms’ demand by using green technological innovations.

Second, environmental information disclosures by buyer enterprise can alleviate financing constraints of seller enterprise through information spillover and enhance seller enterprise’ level of green technological innovations. Environmental information disclosed by buyer firms can serve as a channel for seller firms’ investors to obtain incremental information about seller firms and reduce the risk caused by information asymmetry. Simultaneously, buyer enterprises that transmit high-quality environmental information can play a supervisory role and improve investors’ confidence in seller-firms, thus reducing the financing constraints of seller enterprise and providing them with sufficient funds for investment in green technological innovations.

Third, environmental information disclosure by buyer firms can enhance the level of green technological innovations by raising public attention toward seller firms. As an important stakeholder, buyer-firms’ environmental information disclosure behavior draws public attention to the environmental protection behavior of seller firms through reputation spillover, and promotes green technological innovations of seller-firms through external pressure and internal innovation incentives.

Fourth, environmental information disclosures by buyer firms can enhance seller enterprise’ green technological innovations through improved internal controls. Social responsibility behavior of buyer enterprise can cross corporate boundaries and impact the social responsibility reputation of closely related enterprise. As a business strategy concept, corporate social responsibility helps enterprises meet stakeholders’ expectations by committing to sustainability to reduce the negative impact of the business on the society and the environment. Corporate social responsibility draws managers’ attention to corporate environmental behavior and promotes green technological innovations through internal pressure and internal innovation incentives.

Green technology innovation, as a necessary path to sustainable economic development, is highly valued by the Chinese government. Buyer environmental information disclosure can significantly improve the level of green technology innovation of enterprise. Through the innovation linkage of supply chain enterprise, it can effectively contribute to the realization of green development.

Previous studies have focused on verifying the impact of command-and-control, market-incentive, and voluntary-action environmental regulations on the micro-level of firms. This study focuses on environmental information disclosure. The extant literature has not yet considered the influence that buyer firms, as important external stakeholders of firms, can have on many aspects of firms’ production and operation. This study complements the information asymmetry theory and information spillover effects by analyzing the mediating effects of financing constraints and public concerns on the relation between corporate environmental information and disclosure of corporate green technology innovation.

The following recommendations are made based on the findings of this study.

First, core enterprise in supply chains should be encouraged to actively disclose their environmental information and improve the overall green technological innovations of the supply chain by relying on their relationships with other enterprise. Environmental information disclosures by buyer enterprises can have a spillover effect by driving seller enterprises in a supply chain to implement green technological innovations. China is currently facing increasingly serious environmental problems. Green technology innovation can achieve a win–win situation for both economic benefits and environmental protection. However, enterprise as the main body of green technology innovation are not enough motivation. Government departments should actively explore the incentive system of environmental information disclosure including market instruments. Through tax incentives, special investment, financial subsidies, and other means to stimulate the supply chain core enterprise to actively disclose environmental information. Additionally, tax incentives can guide enterprise to pay attention to the environmental information of supply chain enterprise in the decision-making process to drive the motivation of green technology innovation of other enterprise in the supply chain and realize the green transformation of the supply chain.

Second, standardize the system of environmental disclosure and increase auditing efforts to improve the quality of these disclosures. According to the findings of this paper, buyer’s environmental information disclosure can have an impact on the green technology innovation behavior of seller firms. Moreover, investors of seller firms will obtain more incremental information from the information disclosed by seller firms. Therefore, the quality of environmental information disclosure is directly related to whether the stakeholders can obtain the true and sufficient environmental information to make investment decisions in line with their expectations. In practice, it is found that the quality of environmental information disclosure of Chinese enterprise is low. The government should actively play a guiding role, improve the system of regulations and policies related to environmental information disclosures, unify the standards of environmental information disclosure, and use and strengthen the multifaceted supervision and monitoring. Simultaneously, it should improve the quality of environmental information disclosures, give full play to the information transfer function of environmental information disclosures, and promote the green development of supply chains as a whole.

Third, innovate the environmental information disclosure mode of enterprise and play the role of public supervision. According to the findings of this paper, information spillover from buyer’s environmental information disclosure can increase the public attention and thus influence the green technology innovation of enterprise. Nowadays, it is the era of “Internet+,” and the Internet platform reduces the asymmetry of information owing to its strong connectivity and real-time nature. The establishment of a special environmental information disclosure platform can effectively play the supervisory role of the public. This will also enhance the public’s awareness of environmental protection, strengthen the scope of dissemination and the influence of corporate environmental information, and help achieve the full positive impact of the spillover effect of environmental information disclosures on other stakeholders. Fundamentally enhance the awareness of corporate environmental responsibility. Simultaneously, reflecting and monitoring the environmental performance of enterprise in their production and operation activities through the Internet platform can meet various information needs of different information users.

Finally, when some resource enterprises are sellers, they play a dominant role in their supply chain. When the strong party is the seller enterprise, the seller enterprise reflects the enterprise’s attitude towards environmental responsibility fulfillment by disclosing the quality of environmental information, which will make consumers more willing to buy the enterprise’s products or services in the consumer market, thus increasing the enterprise’s profit. Moreover, the seller enterprise can accurately assess the buyer’s business risk, default risk, and regulatory risk by obtaining the environmental information disclosed by the buyer enterprise. It can screen the buyer enterprises, and when there is a good expectation of the future business condition of the buyer enterprises, it will enhance the motivation of the seller enterprises to strengthen the green technology innovation efforts to meet the buyer’s demand.

Although this study provides compelling insights, it has limitations, too. For example, the enterprise data considered in this study are biased toward the financial data of listed enterprise. At present, Chinese non-listed companies have a certain degree of management oversight. They do not pay as much attention to their own management and social image as listed companies. At the same time, there is less pressure from the external environment. Future research could include a wider range of non-financial information, and the scope of firms could be expanded to include non-listed firms to provide a theoretical basis for more firms to make decisions in this regard. For non-listed companies, if government departments could monitor them more effectively and introduce more effective disciplinary measures, they might be more proactive in disclosing environmental information as listed companies. Public attention to unlisted companies would also serve as a monitoring function.

## Figures and Tables

**Table 1:** Definitions of Variables.

Variable Type	Variable Symbols	Variable Name	Variable Definitions and Interpretation
Explained Variables	TGreen	Corporate Green Patent	Ln (the number of green patent applications of an enterprise in the year + 1)
	IGreen	Corporate Green Invention Patent	Ln (the number of green invention patent applications by an enterprise in the year + 1)
Explanatory variable	EDI	Environmental Information Disclosure	Level of environmental information disclosures by the top five buyers in the previous year weighted by sales share
Control variables	Age	Company Age	Ln (Number of years of business establishment + 1)
	Size	Enterprise size	Ln (Total business assets)
	Lev	Gearing ratio	Total liabilities/Total assets
	Roa	Profitability	Net income/Average total assets at year-end
	Cash	Cash Flow Level	Net cash flow from operating activities/Total assets
	Density	Capital Intensity	Ln (Total fixed assets/Number of employees)
	Indr	Proportion of sole directors	Number of independent directors/Total number of directors in a board
	Share	Management Incentives	Management shareholding/Total corporate share capital
	RD	R&D investment	R&D investment/Operating income
	EFD	External financial dependence index	capital expenditures- cash
Mediated variables	SA	Financing constraints	SA index measures the overall financing constraints of listed companies
	Att	Public attention	Web search index
	ICI	Internal Control	DIB (DIB) Index

**Table 2:** Descriptive statistics.

Variable Name	Sample Size	Average Value	Median	Standard Deviation	Minimum Value	Maximum Value
TGreen	819	0.600	0	0.910	0	6.001
IGreen	819	0.393	0	0.728	0	5.659
EDI	819	1.095	0	1.986	0	14.56
Age	819	14.48	14	5.430	1	32
Size	819	21.79	21.65	1.305	19.34	28.64
Lev	819	0.395	0.379	0.249	0.013	2.613
Roa	819	0.046	0.046	0.102	−1.859	1.202
Cash	819	0.039	0.038	0.063	−0.317	0.273
Density	819	2.251	1.773	1.678	0.305	15.21
Indr	819	0.369	0.333	0.055	0.091	0.714
Share	819	0.185	0.024	0.229	0	0.754
RD	819	2.295	1.816	2.286	0	23.68
EFD	819	0.508	0.501	0.185	0.059	0.941
SA	819	15.70	15.56	1.122	13.46	21.15
Att	665	43.57	46.94	10.07	16.2	59
ICI	819	6.49	6.51	0.179	4.19	6.88

**Table 3:** Correlation test of variables.

	TGreen	IGreen	EDI	Age	Size	Lev	Roa	Cash	Density	Indr	Share	RD	EFD	SA	Att	ICI
TGreen	1															
IGreen	0.853 ***	1														
EDI	0.089 ***	0.129 ***	1													
Age	0.053 *	0.074 **	0.220 ***	1												
Size	0.378 ***	0.374 ***	0.125 ***	0.278 ***	1											
Lev	0.146 ***	0.126 **	0.112 **	0.285 **	0.520 ***	1										
Roa	−0.034 *	−0.036	−0.103 ***	−0.213 ***	−0.134 **	−0.571 ***	1									
Cash	0.107 ***	0.126 ***	0.048	0.068 **	0.135 *	−0.114 ***	0.262 ***	1								
Density	0.122 ***	0.108 ***	0.042	0.183 *	0.401 ***	0.243 ***	−0.225 ***	0.046 *	1							
Indr	−0.024 *	−0.014	−0.056 *	0.059 *	0.0120	−0.362 **	−0.031	0.048	−0.014	1						
Share	−0.148 ***	−0.147 ***	−0.034	−0.258 ***	−0.417 **	−0.421 ***	0.238 **	0.021	−0.259 ***	0.092 **	1					
RD	−0.031	0.004	0.069 **	−0.155 **	−0.322 ***	−0.243 **	0.209 ***	0.027	−0.428 **	0.019	0.178 *	1				
EFD	0.082 **	0.076 **	0.003 ***	0.007 *	0.030 **	0.038 ***	0.084 *	0.017 **	0.044 ***	0.052 **	0.067 *	0.031 ***	1			
SA	0.962 ***	0.232 ***	0.018 **	−0.489 ***	−0.272 ***	−0.153 ***	−0.126 **	−0.028 **	0.017 **	0.155 *	0.148 **	0.014 **	0.110 ***	1		
Att	0.121 ***	0.155 ***	0.244 **	0.138 **	0.292 ***	−0.104 **	−0.228 *	0.081 ***	−0.023 **	0.391 *	0.292 ***	0.017 ***	0.088 **	0.148 *	1	
ICI	0.017 **	0.026 **	0.151 **	0.172 ***	0.251 ***	0.088 *	−0.022 **	0.152	0.195 ***	−0.043	−0.266 **	0.037 *	0.198 ***	0.263	0.210 *	1

Note: ***, **, and * denote significance at the 1%, 5%, and 10% levels, respectively.

**Table 4:** Results of the regression analysis between buyers’ environmental information disclosures and corporate green technological innovations.

Variable	Model (1)	Model (1)
	TGreen	IGreen
EDI	0.0477 **	0.0524 ***
	(2.1426)	(2.7328)
Age	−0.0265	0.0140
	(−0.2938)	(0.1973)
Size	0.3123 ***	0.2461 ***
	(8.5841)	(8.0129)
Lev	−0.2017	−0.3557 **
	(−1.1120)	(−2.3949)
Roa	−1.4360 **	−1.6336 ***
	(−2.1742)	(−2.8435)
Cash	1.1548 **	1.1920 ***
	(2.5716)	(3.4293)
Density	−0.0109	−0.0131
	(−0.2953)	(−0.4343)
Indr	−0.8384	−0.6524
	(−1.5988)	(−1.4714)
Share	−0.1767	−0.1712 *
	(−1.3234)	(−1.7866)
RD	0.0652 ***	0.0417 ***
	(3.5671)	(3.2675)
EFD	0.0612 **	0.0391 **
	(2.4526)	(2.1388)
Constant	−4.9513 ***	−3.8763 ***
	(−6.4217)	(−6.0421)
Year Fx	yes	yes
Industry Fx	yes	yes
N	819	819
R^2^	0.2758	0.2932

Note: ***, **, and * denote significance at the 1%, 5%, and 10% levels, respectively, with *t*-test values in parentheses.

**Table 5:** Mediating effects of financing constraints.

Variable	Model (1)	Model (2)	Model (3)	Model (1)	Model (3)
	TGreen	SA	TGreen	IGreen	IGreen
EDI	0.0477 **	0.0069 **	0.0388 *	0.0524 ***	0.0436 **
	(2.1426)	(2.3157)	(1.9084)	(2.7328)	(2.4143)
SA			1.1023 ***		1.2386 ***
			(2.6563)		(3.3062)
Age	−0.0265	−0.4968 ***	0.5214 **	0.0140	0.6315 ***
	(−0.2938)	(−33.3224)	(2.2862)	(0.1973)	(3.1729)
Size	0.3123 ***	−0.0285 ***	0.3358 ***	0.2461 ***	0.2818 ***
	(8.5841)	(−4.3323)	(8.5470)	(8.0129)	(8.1392)
Lev	−0.2017	−0.0036	−0.2046	−0.3557 **	−0.3541 **
	(−1.1120)	(−0.0672)	(−1.1415)	(−2.3949)	(−2.5214)
Roa	−1.4360 **	−0.1237	−1.3231 **	−1.6336 ***	−1.4917 ***
	(−2.1742)	(−1.2293)	(−2.0944)	(−2.8435)	(−2.7827)
Cash	1.1548 **	−0.0519	1.1929 ***	1.1920 ***	1.2429 ***
	(2.5716)	(−0.6559)	(2.7313)	(3.4293)	(3.7214)
Density	−0.0109	0.0163 ***	−0.0285	−0.0131	−0.0348
	(−0.2953)	(3.5832)	(−0.7865)	(−0.4343)	(−1.1192)
Indr	−0.8384	0.1735 *	−1.0198 **	−0.6524	−0.8543 *
	(−1.5988)	(1.7124)	(−1.9869)	(−1.4714)	(−1.9594)
Share	−0.1767	0.0487 ***	−0.2298 *	−0.1712 *	−0.2295 **
	(−1.3234)	(2.7283)	(−1.6872)	(−1.7866)	(−2.3749)
RD	0.0652 ***	0.0047 **	0.0598 ***	0.0417 ***	0.0485 ***
	(3.5671)	(2.2995)	(3.2876)	(3.2675)	(2.9395)
EFD	0.0612 **	0.0443 **	0.0638 ***	0.0391 **	0.0472 ***
	(2.4526)	(2.2473)	(2.2869)	(2.1388)	(2.4785)
Constant	−4.9513 ***	−1.8978 ***	−2.8693 ***	−3.8763 ***	−1.5293 **
	(−6.4217)	(−13.5328)	(−3.0897)	(−6.0421)	(−2.0184)
Year Fx	yes	yes	yes	yes	yes
Industry Fx	yes	yes	yes	yes	yes
N	819	819	819	819	819
R^2^	0.2758	0.8298	0.2896	0.2932	0.321

Note: ***, **, and * denote significance at the 1%, 5%, and 10% levels, respectively, with *t*-test values in parentheses.

**Table 6:** Mediating effect of public attention.

Variable	Model (1)	Model (4)	Model (5)	Model (1)	Model (5)
	TGreen	Att	TGreen	IGreen	IGreen
EDI	0.0438 **	0.0289 ***	0.0469 *	0.0564 ***	0.0474 **
	(2.1025)	(2.6428)	(1.8639)	(2.5987)	(2.2496)
Att			0.1493 *		0.2228 ***
			(1.9269)		(3.4892)
Age	−0.0027	0.1091 **	−0.0464	0.0272	−0.0233
	(−0.0183)	(2.0113)	(−0.4047)	(0.3295)	(−0.2554)
Size	0.3046 ***	0.2945 ***	0.2565 ***	0.2563 ***	0.1868 ***
	(7.7198)	(14.4681)	(5.7983)	(7.4460)	(5.0474)
Lev	−0.2499	−0.1093	−0.1978	−0.4346 **	−0.3592 **
	(−1.2089)	(−0.9170)	(−0.9147)	(−2.5120)	(−2.0241)
Roa	−1.4776 **	−1.0292 *	−1.3971 *	−1.7533 ***	−1.6442 **
	(−1.9997)	(−1.9281)	(−1.7789)	(−2.7561)	(−2.4736)
Cash	1.0774 **	0.9864 ***	0.9656 *	1.1093 ***	0.9794 **
	(2.2797)	(2.7796)	(1.8762)	(2.9577)	(2.4457)
Density	−0.0197	−0.0262	−0.0253	−0.0396	−0.0276
	(−0.4789)	(−0.9047)	(−0.4776)	(−0.8772)	(−0.7765)
Indr	−1.0299 *	0.3931	−1.3193 **	−0.7389	−1.0442 **
	(−1.8291)	(1.2392)	(−2.29687)	(−1.5533)	(−2.1938)
Share	−0.1992	−0.2945 ***	−0.2262	−0.1989 *	−0.1897 *
	(−1.3594)	(−3.2799)	(−1.4399)	(−1.8738)	(−1.6875)
RD	0.0588 ***	0.0181	0.0584 ***	0.0497 ***	0.0478 ***
	(2.9437)	(1.5639)	(2.8379)	(2.7789)	(2.7448)
EFD	0.0609 ***	0.0572 *	0.0529 **	0.0617 ***	0.0593 **
	(2.2728)	(2.4396)	(2.6399)	(2.1938)	(2.7389)
Constant	−4.4695 ***	5.9662 ***	−5.0895 ***	−3.5197 ***	−4.6181 ***
	(−5.1593)	(12.2264)	(−4.8343)	(−4.8966)	(−5.3452)
Year Fx	yes	yes	yes	yes	yes
Industry Fx	yes	yes	yes	yes	yes
N	665	665	665	665	665
R^2^	0.2789	0.5545	0.2881	0.2997	0.3262

Note: ***, **, and * denote significance at the 1%, 5%, and 10% levels, respectively, with *t*-test values in parentheses.

**Table 7:** Internal controls’ mediating effects.

Variable	Model (1)	Model (6)	Model (7)	Model (1)	Model (7)
	TGreen	ICI	TGreen	IGreen	IGreen
EDI	0.0477 **	0.0094 **	0.0778 *	0.0524 ***	0.0786 **
	(2.1426)	(2.4785)	(1.4561)	(2.7328)	(2.2196)
ICI			0.9360 ***		0.9468 ***
			(2.3279)		(3.1565)
Age	−0.0265	−0.4796 **	0.4248 **	0.0140	0.4660 **
	(−0.2938)	(−6.3374)	(2.1486)	(0.1973)	(3.1879)
Size	0.3123 ***	−0.0324 ***	0.6716 ***	0.2461 ***	0.3598 ***
	(8.5841)	(−4.4887)	(8.7260)	(8.0129)	(8.1697)
Lev	−0.2017	−0.0136	−0.4290	−0.3557 **	−0.3162 **
	(−1.1120)	(−0.0396)	(−1.0763)	(−2.3949)	(−2.2642)
Roa	−1.4360 **	−0.2391	−1.6778 **	−1.6336 ***	−1.9834 ***
	(−2.1742)	(−1.1236)	(−2.1928)	(−2.8435)	(−2.3897)
Cash	1.1548 **	−0.0876	1.3867 **	1.1920 ***	1.4728 ***
	(2.5716)	(−0.3274)	(2.3681)	(3.4293)	(3.3671)
Density	−0.0109	0.0396 **	−0.0583	−0.0131	−0.0476
	(−0.2953)	(3.2886)	(−0.5979)	(−0.4343)	(−1.0693)
Indr	−0.8384	0.3283 *	−1.0496 *	−0.6524	−0.7326 *
	(−1.5988)	(1.3582)	(−1.6889)	(−1.4714)	(−1.6797)
Share	−0.1767	0.0994 **	−0.4576 *	−0.1712 *	−0.3776 **
	(−1.3234)	(2.3678)	(−1.3462)	(−1.7866)	(−2.1894)
RD	0.0652 ***	0.0176 **	0.1242 **	0.0417 ***	0.0932 ***
	(3.5671)	(2.1596)	(3.1476)	(3.2675)	(2.8778)
EFD	0.0612 **	0.0595 *	0.0607 **	0.0391 **	0.0462 **
	(2.4526)	(2.4436)	(2.3749)	(2.1388)	(2.1698)
Constant	−4.9513 ***	−1.8693 ***	−2.7381 **	−3.8763 ***	−1.1588 ***
	(−6.4217)	(−13.3862)	(−3.1795)	(−6.0421)	(−2.1277)
Year Fx	yes	yes	yes	yes	yes
Industry Fx	yes	yes	yes	yes	yes
N	819	819	819	819	819
R^2^	0.2758	0.6176	0.4792	0.2932	0.3468

Note: ***, **, and * denote significance at the 1%, 5%, and 10% levels, respectively, with *t*-test values in parentheses.

**Table 8:** Results of the regression analysis of the effect of environmental information disclosures by buyer firms on the number of green patents granted by buyer firms.

Variable	AthrTGreen	AthrIGreen
EDI	0.0497 **	0.0572 ***
	(2.5497)	(2.9283)
Age	−0.0214	0.0462
	(−0.0316)	(0.8995)
Size	0.2493 ***	0.1562 ***
	(7.5797)	(6.5169)
Lev	−0.3178 *	−0.3942 ***
	(−1.9792)	(−3.2693)
Roa	−1.7496 **	−1.4129 ***
	(−2.5485)	(−2.9045)
Cash	0.2446	0.4494
	(0.5299)	(1.3653)
Density	−0.0114	0.0181
	(−0.0549)	(0.4598)
Indr	0.0638	0.4795
	(0.1092)	(1.1761)
Share	−0.2046	−0.1494
	(−1.6384)	(−1.5597)
RD	0.0392 **	0.0246 **
	(2.2691)	(2.1689)
EFD	0.0491 **	0.0396 **
	(2.7638)	(2.6649)
Constant	−4.7622 ***	−3.0431 ***
	(−6.4799)	(−5.8781)
Year Fx	yes	yes
Industry Fx	yes	yes
N	819	819
R^2^	0.2573	0.2486

Note: ***, **, and * denote significance at the 1%, 5%, and 10% levels, respectively, with t-test values in parentheses.

## Data Availability

The raw data supporting the conclusions of this article will be made available by the authors, without undue reservation.
